# Potential roles of circulating microRNAs in the healing of type 1 diabetic wounds treated with green tea extract: molecular and biochemical study

**DOI:** 10.1016/j.heliyon.2023.e22020

**Published:** 2023-11-03

**Authors:** Hadeel A. Al-Rawaf, Sami A. Gabr, Ahmad H. Alghadir

**Affiliations:** a Rehabilitation Research Chair, Department of Rehabilitation Sciences, College of Applied Medical Sciences, King Saud University, Riyadh, Saudi Arabia; b Department of Clinical Laboratory Sciences, College of Applied Medical Sciences, King Saud University, Riyadh, Saudi Arabia

**Keywords:** Diabetic wounds, Camellia sinensis, Circulating miRNAs, apoptosis, Polyphenols, PCR, HPLC

## Abstract

**Background:**

Circulating miRNAs have been implicated in various aspects of diabetic wound healing, including inflammation, angiogenesis, and extracellular matrix remodeling. Thus, in alternative herbal medicine strategies, miRNAs will be potential therapeutic molecular targets in nonhealing wounds. These could be valuable elements for understanding the molecular basis of diabetic wound healing and could be used as good elements in bioinformatics.

**Objectives:**

To elucidate the molecular mechanisms of microRNAs in association with apoptosis-inducing genes in controlling skin wound healing in diabetic wounds treated with green tea polyphenols (GTPs).

**Methods:**

Green tea hydro extract (GTE) at doses of100–200 mg/ml was topically applied to the skin tissues of rats with T1DM induced by a single dose of streptozotocin (STZ; 100 mg/kg, in 0.01 M sodium citrate, pH 4.3–4.5) injected intraperitoneally for seven consecutive days to induce T1DM. The rats were treated with green tea for three weeks. A sterile surgical blade was used to inflict a circular wound approximately 2 cm in diameter on the anterior-dorsal side of previously anesthetized rats by a combination of ketamine hydrochloride (50 mg/kg, i.e., body weight) and xylazine hydrochloride. Afterward, the molecular roles of the circulating miRNAs miR-21, miR-23a, miR-146a, and miR-29b and apoptotic genes were determined by quantitative real-time PCR to evaluate Bax, Caspase-3, and Bcl-2 in wound healing. In addition, HPLC analysis was also performed to estimate the active polyphenols (GTPs) present in the hydro extract of green tea leaves.

**Results:**

Wound healing was improved in diabetic skin wounds following treatment with GTE at doses of 100–200 mg/dl for three weeks. The wound parameters contraction, epithelialization, and scar formation significantly improved in a short time (14 days) compared to the longer periods identified in diabetic non-treated rats (20 days) and the standard control (15.5 days). Molecular analyses reported a significant increase in the levels of miR-21, miR-23a, and miR-146a and a decrease in the levels of miR-29b in green tea-treated diabetic rats compared to those in the standard control and STZ-diabetic non-treated rats. In addition, the molecular apoptotic genes Bax and caspase-3 significantly increased, and the BcL-2 gene significantly decreased following treatment with green tea polyphenols.

**Conclusions:**

The data showed that active green tea polyphenols (GTPs) present in GTE significantly improved diabetic wound healing by controlling apoptotic genes and the circulating microRNAs miR-21, miR-23a, miR-146a, and miR-29b, which might be involved in cellular apoptosis and angiogenesis processes. Thus, to establish a future model for the treatment of diabetic wounds, further studies are needed to understand the potential association of these biological parameters with the wound-healing process in diabetic wounds.

## 1 Introduction

Diabetes is one of the most serious diseases that affects human health globally. Clinically, diabetes is associated with many cellular, physiological, and metabolic disorders, particularly in the healing of skin wounds, which significantly impair patient quality of life [[1], [2], [3], [4], [5], [6]].

In diabetic wounds, different biological processes, such as inflammatory (homeostasis and inflammation), proliferative (granulation, contraction, and epithelialization), and tissue remodeling (strength and scar formation) phases, which are needed for normal wound healing, are significantly suppressed by increased cellular sugar [[7], [8], [9], [10], [11], [12], [13], [14]].

MicroRNAs (miRNAs) are a class of small noncoding RNA molecules that play a critical role in regulating gene expression at the posttranscriptional level, making them important regulators of many biological processes, including wound healing. In the context of diabetic wound healing, miRNAs have been shown to play a significant role in regulating various cellular processes that are essential for proper wound healing [[15], [16], [17], [18], [19], [20], [21]]. In addition, circulating miRNAs alleviate diabetic effects via the regulation of insulin synthesis and other cellular-related organelles, such as β‐cells, β‐cell fate, islet mass formation, cellular membrane electrical excitability (ATP:ADP ratio), and insulin granule exocytosis [22].

In individuals with diabetes, impaired wound healing is a major complication leading to chronic wounds and even amputations. miRNAs have been implicated in various aspects of diabetic wound healing, including inflammation, angiogenesis, and extracellular matrix remodeling. For example, hypoxy-miRs such as miR-210, miR-21, and miR-203 were expressed in association with the progression of ischemia/hypoxia in non-healed diabetic wounds [[23], [24], [25]]. In addition, miR-21, miR-126, and miR-29 have been shown to promote wound healing by enhancing angiogenesis, reducing inflammation, and inhibiting extracellular matrix deposition, which can impair wound healing in individuals with diabetes [[23], [24], [25]]. Additionally, miRNAs such as miR-21, miR-23a, and miR-146a play a significant role in regulating and accelerating the complex cellular processes involved in diabetic wound healing, such as keratinocyte migration, re-epithelialization, cellular apoptosis, proliferation, and angiogenesis [[26], [27], [28], [29], [30], [31]].

Thus, the role of miRNAs in the healing of diabetic wounds is important because they have been shown to play a critical role in regulating gene expression and various cellular processes that are essential for wound healing, including inflammation, angiogenesis, and tissue repair [[23], [24], [25], [26], [27], [28], [29], [30], [31]]. In addition, miRNAs have been shown to be dysregulated in diabetic wounds, which in turn can contribute to impaired healing processes and might be regulated by phytochemicals, particularly in cellular development, proliferation, differentiation, and apoptosis [32].

Green tea (Camellia sinensis, Theaceae family) has been used in different traditional trials of human health as an antioxidant, anti-inflammatory, antimicrobial, and antidiabetic agent [[33], [34], [35]].

The presence of high levels of polyphenolic compounds, particularly catechins, can help reduce oxidative stress and inflammation as well as improve blood flow to the wound site. Thus, green tea has been studied as a potential agent for promoting the healing of diabetic wounds by the production of cellular collagen and fibronectin fibrils with an increase in nitric oxide. In addition, the application of green tea as an antihealing agent promotes angiogenesis by molecularly controlling the expression of circulating hypoxia-responsive microRNAs [36].

While long noncoding RNAs (lncRNAs) and circulating microRNAs (miRNAs) are both noncoding RNA molecules that have been investigated as potential biomarkers for various diseases, including diabetic wound healing, there are some differences in their utility as biomarkers. For example, lncRNAs may provide more specific information about the molecular mechanisms involved in wound healing, while circulating miRNAs may be more useful as general indicators of disease progression [[37], [38], [39]]. In addition, although many studies have investigated the effects of herbal remedies on diabetic wound healing and the potential role of lncRNAs in these processes, no or few therapeutic trials have specifically investigated the use of lncRNAs as biomarkers in herbal remedies to improve diabetic wound healing [[40], [41], [42]].

Therefore, we hypothesized that circulatory microRNA molecules may serve as useful biomarkers and good targets for predicting wound healing outcomes in therapeutic interventions based upon a hydro extract of green tea leaves. Thus, in this study, we aimed to evaluate the potential role of the circulating microRNAs miR-21, miR-23a, miR-146a, and miR-29b and their correlation with the cellular apoptotic markers Bax, caspase-3, and the BcL-2 gene in wounds of diabetic and nondiabetic rats treated with a hydro extract of green tea leaves.

## 2 Materials and methods

### 2.1 Materials

#### 2.1.1 Green tea

Samples of green tea (*Camellia sinensis*, Theaceae) plants (NCBI: txid4442) were purchased from a convenience store (Othaim Markets) in Riyadh, KSA.

#### 2.1.2 Catechin and caffeine standards

HPLC grade standards of green tea catechins (GTCs), (+)-catechin, (−)-epicatechin, (−)-epigallocatechin, (−)-epicatechin gallate, (−)-epigallocatechin gallate, (−)-gallocatechin gallate, and caffeine were purchased from Sigma Company (Saint-Louis, MO, USA) [36,43].

### 2.2 Methods

#### 2.2.1 Green tea extraction and HPLC analysis

A total of 20 gm of green tea leaves with a ratio of 1/50 tea/water was soaked in 1 L of a-heated water (ddH_2_O) for 10 min at 80 °C as previously reported [[44], [45], [46]]. The tea leaves were soaked three times for 10 min each, and the extracted solution was collected each time. Then, a total of 1.5 mL of the green tea extract was used to estimate the concentrations of green tea catechins (GTCs) as mentioned previously [[44], [45], [46]]. All green tea extracts were first filtered by using a 0.20 μm filter (Aerodisc LC13 PVDF, Gelman Laboratory, Ann Arbor, MI) and diluted with ddH2O HPLC grade water. Then, GTC concentrations were estimated in all green tea extracts by the reversed-phase high-performance liquid chromatography (RP-HPLC) method as mentioned previously [[44], [45], [46]].

#### 2.2.2 Animals and study design

The study was performed on a total of 50 healthy ten-week-old male Wistar rats weighing 150–180 g were selected from the animal house of the experimental animal care center, College of Applied Medical Sciences, King Saud University, Riyadh, Saudi Arabia [[47], [48], [49]]. The animals were randomly assigned to five groups of 10 each. Then, they were housed individually in cages and bred on normal feeding and drinking in a 12 h light/dark cycle at a controlled temperature of 24 °C. The research design applied in our study meets the Animal Research: Reporting of In Vivo Experiments (ARRIVE) guidelines. All the experimental procedures were carried out according to the Guidelines for the Care and Use of Laboratory Animals established by the National Institutes of Health of the United States (NIH) publication (No. 85-23, 1996). The protocol of the study was approved by the institutional animal ethical committee, Rehabilitation Research Chair (RRC), College of Applied Medical Sciences, King Saud University, Riyadh, Saudi Arabia, under file number [ID: RRC-2019-071].

#### 2.2.3 Induction of type 1 diabetes (T1DM)

Rats were injected intraperitoneally with a single dose of streptozotocin (STZ; 100 mg/kg, in 0.01 M sodium citrate, pH 4.3–4.5) for seven consecutive days to induce T1DM as previously mentioned [[49], [50], [51]]. The use of STZ at a dose of 100 mg/kg attempts to promote direct toxicity to ***β*** cells with little or no measurable insulin production. The induction of the T1DM model was considered successfully established in rats when FBG levels were higher than 250 mg/dl [[49], [50], [51]].

Control rats were injected only with a saline vehicle. Following 7 days, a Hitachi 7060C automatic colorimeter was used to estimate fasting blood sugar (FBG) in control and STZ-treated rats. In addition, serum insulin and C-peptide were estimated in all rats by using the Sandwich immune ELISA [[49], [50], [51]]. Additionally, a Glycomet kit (Biocode Hycel, Massy, France) was used to quantify hemoglobin A1c (HbA1c) levels in the whole blood samples using exchange chromatography. To estimate the antidiabetic activity of green tea extracts (GTEs), STZ-induced type 1 diabetic rats received green tea extracts (GTEs) orally at doses of 100 and 200 mg/ml for three weeks. Finally, the results were compared to those with control and diabetic rats.

#### 2.2.4 Excision wound assessments

Before assessing wounds, the rats were anesthetized by a combination of ketamine hydrochloride (50 mg/kg, i.e., body weight) and xylazine hydrochloride (10 mg/kg, body weight). Then, the rats were shaved at a predetermined area in the dorsum portion using depilatory cream (Reckitt Benckiser, Inc., UK). For each rat, a circular wound of approximately 2 cm in diameter was inflicted on the anterior-dorsal side by using a sterile surgical blade as mentioned previously [52].

#### 2.2.5 Wound Treatments

Rats were classified into five groups: G1 (control): healthy rats treated with a saline vehicle twice daily for three consecutive weeks; G2 (DM group): diabetic non-treated rats received a saline vehicle twice daily for three weeks; G3 (Standard control): diabetic rats treated topically with 0.2 mL Intrasite gel twice daily as a reference standard control. Wounds of rats in G4 and G5 were topically treated with 0.2 mL of 100 (low dose) and 200 (high dose) mg/ml of a hydro extract of green tea in-vehicle, respectively, twice daily for three consecutive weeks. The application of Camellia sinensis showed high potential in wound healing activity, particularly at higher concentrations [53]. A controlled camera (Sony Cyber-Shot, Dscw80) was used to monitor the progression of wound contraction, epithelialization, and complete wound healing on the baseline, 3rd, 6th, 9th, 15th, and 21st day of wounding treatment using green tea extracts [53]. For each group, contraction in the wound area was evaluated by using the ImageJ program. In addition, the epithelialization period of the treated wounds refers to the total days needed for falling off the dead wound tissue remnants without leaving any residual raw wound, as mentioned previously [54]. Finally, the percent wound contraction was evaluated according to the following equation [55,56].

{ % of wound closure = {(A0 – At)/A0] x 100}

where A0 is the original wound area and at is the area of the wound at the time of biopsy.

#### 2.2.6 Assessment of microRNAs from SkinWounds

##### 2.2.6.1 Extraction and Purification of circulating RNA

RNA was extracted from all skin wound tissues of control, treated, and untreated diabetic rats by using TRIzol reagent (Clontech Laboratories Inc., Mountain View, CA, USA) as previously reported [49,57]. The integrity and quantity of the extracted total RNA were measured by using an Agilent 2100 Bioanalyzer (Agilent Technologies) [58]. Moreover, cDNA templates for miR-21, miR-23a, miR-146a, and miR-29 b were synthesized from the extracted purified RNA by using the Mir-X miRNA First-Strand Synthesis Kit (Clontech Laboratories Inc.) as mentioned previously [49,57].

##### 2.2.6.2 Real-time qPCR analysis of microRNAs

A quantitative Real-Time PCR System (Applied Biosystems 7300, Foster City, CA, USA) and Mir-X miRNA qRT‒PCR SYBR Kit (Clontech Laboratories Inc.) were used to identify the expression of the circulating miRNAs miR-21, miR-23 a, miR-146 a, and miR-29 b in skin wound tissues of green tea-treated and untreated rats [49,50]. In this experiment, ready-made solutions containing the primers and probes for miR-21, miR-23 a, miR-146 a, and miR-29b (ABI, Applied Biosystems, Foster City, CA) (Table 1) were mixed with cDNA templates and subjected to different amplifying PCR cycles [59]. The levels of the analyzed miRNAs were measured according to a normalized internal quantitative control, U6 snRNA levels, and the 2−ΔΔCt system [59]. To avoid errors and to have exact results, both amplified miRNAs and endogenous controls will run in duplicate to determine the values of the cycle threshold mean [49,60].

**Table 1:** list of primer sequences used in real-time PCR analysis.

Gene	Primer sequences
***GAPDH***	For 5′-CAA GGT CAT CCA TGA CAA CTT TG-3′ Rev 5′-GTC CAC CAC CCT GTT GCT GTA G-3′
**Bax**	For 5′-GTC GCC CTT TTC TAC TTT GCC -3′ Rev 5′-CTC CCG CCA CAA AGA TGG TCA-3′
**Bcl-2**	For 5′-CCC CTC GTC CAA GAA TGC AA-3′ Rev 5′-TCT CCC GGT TAT CGT ACC CTG-3′
**Caspase-3**	For 5′-GGTATTGAGACAGACAGTGG-3′ Rev 5′-CATGGGATCTGTTTCTTTGC-3′
**MiR-146a**	For 5′- ACACTCCAGCTGGGTGAGAACTGAATTCCATG -3′ Rev 5′- TGTCGTGGAGTCGGCAATTC-3′
**miR-21**	For: 5′-CGGCGGTAGCTTATCAGACTGATGT-3′ Rev: 5′-GTGCAGGGTCCGAGGT-3′
**miR-23a**	For 5′- GCGCATCACATTGCCAGGG-3′ Rev 5′- CAGTGCAGGGTCCGAGGT-3′
**miR-29 b**	For 5′- CTAGCCTGCAGGATTGTCATTGTCTTGAACAA-3′ Rev 5′- ATCCGGCCGGCCGTGAATGAAGCAGTCCTCCA-3′

##### 2.2.6.3 Assessment of apoptotic genes from skin wounds

A reverse Transcriptase Kit (Thermo Fisher Scientific, MA, USA) was used to synthesize cDNA for each respective apoptotic gene from the skin wound tissues of green tea-treated and non-treated rats as previously reported [61]. In the PCR analysis test, a SYBR green dye universal master mix (Bioron GmbH, Germany) in addition to primer sequences for apoptosis (Bax, Bcl-2, and caspase-3) (Sigma‒Aldrich, Thermo Scientific, Germany) (Table 1) was used to amplify the synthesized cDNA for 40 cycles as mentioned previously [61]. The average copy number of the resultant PCR components was normalized according to GAPDH, which is used as an internal housekeeping gene [61].

#### 2.2.7 Statistical analysis

The data of the present study were analyzed by using the statistical software SPSS version 17. The results of all groups are expressed as the mean and standard deviation [49]. Moreover, the comparisons of mean values of the studied variables, such as miRNAs, apoptotic markers, bcl-2, bax, caspase-3, and diabetic control parameters, were identified by using Kruskal–Wallis one-way ANOVA and post hoc (Tukey HSD) test, respectively. Additionally, Spearman rank correlation analysis was performed to assess the relationship between various study parameters [49]. The data obtained were deemed significant at P < 0.05.

## 3 Results

### 3.1 HPLC analysis of green tea polyphenols (GTPs)

**Fig. 1:**
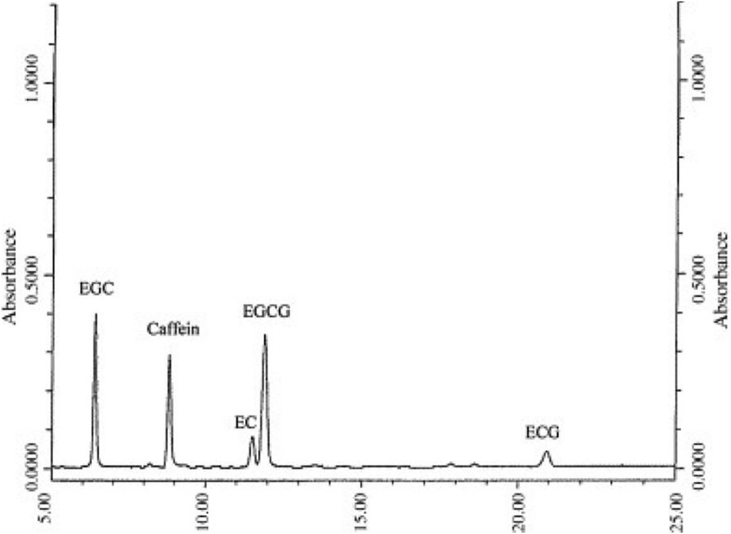
Chromatogram of green tea chatichens (GTCs) of green tea leaves soaked in ddH_2_O water preheated at 80 °C for 10 min. EGC: (−)-epigallocatechin, EC: (−)-epicatechin, EGCG: (−)-epigallo-catechine gallate, ECG: (−)-epicatechin gallate, and Caffein.

Five active polyphenols (GTPs) with approximately 76.6 % of the total active components were identified in green tea extract (GTE) according to their respective retention times during HPLC analysis, as shown in Table (2) and Figure (1). The results showed that EGCG was identified in GTE in large amounts (42.9 %), followed by EGC (12.0 %), EC (10.5 %), ECG (7.1 %), and caffeine (4.1 %), as shown in Table 2.

**Table 2:** Concentrations (in μg/mL) of green tea catechins (GTCs) and caffein in green tea hydro extract (20 g/1L ddH_2_O).

Compound	Concentration	Percentage (%)
**EGCG**	525.0 ± 56.6	42.9
**EGC**	448.2 ± 21.8	12.0
**EC**	62.7 ± 6.1	10.5
**ECG**	56.4 ± 9.3	7.1
**Caffein**	212.2 ± 6.3	4.1
		Total = 76.6 %

EGCG: (−)-epigallo-catechine gallate, EGC: (−)-epigallocatechin, EC: (−)-epicatechin, ECG: (−)-epicatechin gallate.

### 3.2 Green tea polyphenols (GTPs) ameliorate diabetes in STZ-induced T1DM rats

Table (3) shows the effects of green tea extracts at doses of 100 and 200 mg/ml on T1DM induced by intraperitoneal injection of STZ in a single dose (100 mg/kg) for seven days. In this experiment, FBS, HbA1c, and C-peptide, along with body weight, were evaluated as a measure of diabetes (Table 3). Compared to those in the normal control group (G1), rats with T1DM (G2) showed a significant (P < 0.05) increase in the levels of FBS, HbA1C, and C-peptide with a reduction in the levels of insulin and body weights. Moreover, rats treated with green tea extracts at a dose of 100 or 200 mg/ml showed an improvement in the diabetic parameters FBS, HbA1c, C-peptide, insulin, and body weight (p = 0.001) compared to those in diabetic nontreated T1DM rats. Similarly, the diabetic parameters FBS, HbA1c, C-peptide, insulin, and body weight were significantly improved in T1DM rats (G3) treated with the reference standard control (0.2 mL Intrasite gel) compared to diabetic nontreated T1DM rats (Table 3).

**Table 3:** Anti-diabetic effects of hydro green tea extract following STZ-induced a stablished T1DM diabetes in rats.

Variables	G1	G2	G3	G4	G5
**Body weight (g)**	42.5 ± 4.2	25.5 ± 2.8^a^	28.2 ± 1.8^a^	31.4 ± 5.4^b^	36.2 ± 3.5^c^
**FBG (mg/dl)**	92.7 ± 3.2	315 ± 6.8^a^	258 ± 3.8^a^	239.5 ± 6.5^b^	186.7 ± 4.5^c^
**HbA1C**	3.1 ± 1.5	9.7 ± 2.4^a^	8.5 ± 1.4^a^	4.9 ± 2.1^b^	3.8 ± 1.2^c^
**Insulin (mU/L)**	9.6 ± 1.8	3.5 ± 1.2^a^	4.9 ± 2.3 a	7.5 ± 1.5^b^	8.4 ± 1.9^c^
**C-peptide (ng/mL)**	0.52 ± 0.45	3.75 ± 0.82^a^	3.0 ± 0.89^a^	2.1 ± 0.75^b^	1.6 ± 0.81^c^

Data are expressed as mean ± SD; ^a^ p < 0.05 (compared with control), ^b^ p < 0.01 (G3 vs. control or diabetic group), and ^c^ p < 0.001 (diabetic group vs. GTE; 100 mg/ml (Q4)/or 200 mg/ml (G5)). FBG: fasting blood sugar, and HbA1C: glycated hemoglobin A1c. G1 (Control), G2((diabetic), G3 (standard control; 0.2 mL Intrasite gel), G4 (GTE at 100 mg/ml), and G5 (GTE at 200 mg/ml).

### 3.3 Treatments of T1DM diabetic skin wounds

**Fig. 2:**
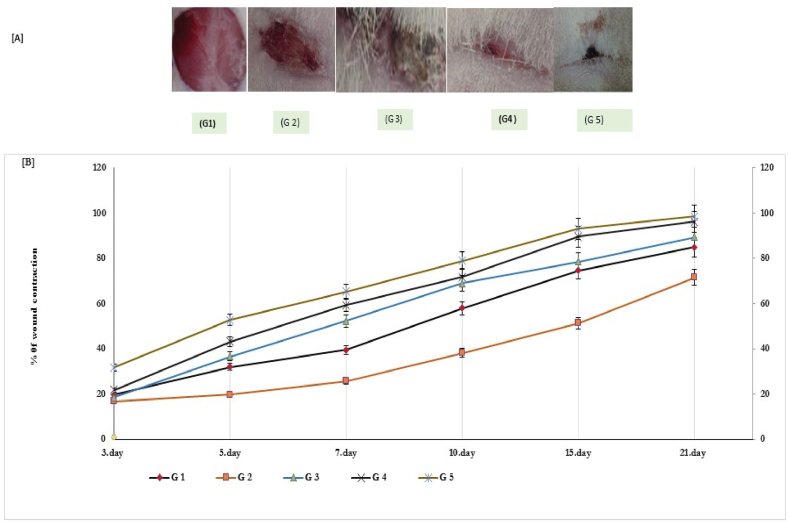
wound closer rates at different time intervals in diabetic and non-diabetic rats treated with green tea extract for 21 days. In figure (2a &2B), diabetic rats treated with green tea extract (GTE) at doses of 100 mg/ml (G4) and 200 mg/ml (G5) showed significant improvement in wound closer (≥90 %) compared to control group (G1), diabetic (G2), and standard control drug (G3) respectively. G1 (Control), G2 ((diabetic), G3 ((Standard control), G4 (GTE at 100 mg/ml), and G5 (GTE at 200 mg/ml).-

Treatments of diabetic skin wounds with green tea extract at doses of 100 mg/ml and 200 mg/ml significantly improved wound contraction, epithelialization, and scar formation in the diabetic excision wound model, as shown in Table (4) and Figure (2).

In nontreated diabetic wounds (G2), a delay in wound healing was reported compared to that reported in the (p < 0.05) control group. Regular follow-up showed that complete wound closure, epithelialization, and scar formation were significantly reported in diabetic wounds on Day 20, with a low average closure rate of 53.5 %, while most of the wounds were unhealed completely, as shown in Table (2) and Fig. 2 (A).

In addition, T1DM diabetic wounds treated with green tea extracts at doses of 100 mg/ml and 200 mg/ml (G4 & G5) showed complete wound closure, epithelialization, and scar formation in short periods (11–14 days) compared (P < 0.001) to healing parameters measured in the standard control (G3), diabetic (G2), and normal control groups (G1) (Table 4) and Figure (2 A & 2B). The treatment of T1DM diabetic wounds with GTE at respective doses significantly improved wound closure (94%–97 %) with a maximum scar formation (≥96.4 %), which significantly increased the healing effect with a ratio over (≥40 %) compared to that of diabetic wounds (Table 4).

**Table 4:** Effect of green tea polyphenols (GTPs) on wound contraction and epithelialization in diabetic excision wound model.

Group			Wound contraction					Scar area (mm^2^)	Epithelialization period (days)
	Baseline	3rd day	6th day	9th day	15th day	21st day	%		
G1	192.3 ± 0.74	180 ± 0.52	171.1 ± 1.49	98.6 ± 2.8	88.9 ± 3.6	86.3 ± 5.4	75 %	92.6 ± 2.7	16.9 ± 0.85
G2	195.2 ± 0.42	192.3 ± 0.89^a^	185.8 ± 1.6^a^	146.2 ± 3.6^a^	139.1 ± 2.5^a^	135.7 ± 3.2^a^	53.5 %	65.7 ± 3.2^a^	19.5 ± 0.75^a^
G3	196.5 ± 0.35	186.5 ± 0.74^b^	165.9 ± 1.4^c^	115.3 ± 2.4^b^	105.4 ± 1.5^b^	101.4 ± 2.5^b^	81.5 %	94.8 ± 2.8^b^	15.5 ± 0.53^c^
G4	194.6 ± 0.65	178.4 ± 0.42^c^	116.7 ± 2.9^c^	79.8 ± 3.1^c^	71.5 ± 2.7^c^	69.7 ± 3.7^c^	93.5 %	96.4 ± 1.8^c^	13.6 ± 0.78^c^
G5	196.3 ± 0.85	165.9 ± 0.95^c^	89.5 ± 2.5^c^	48.8 ± 2.6^c^	41.8 ± 3.7^c^	39.4 ± 4.7^c^	96.5 %	98.4 ± 1.6^c^	10.8 ± 1.6^c^

^a^P < 0.05 (G2 vs G1), ^b^P < 0.01 (G3 vs G1 or G2), and ^c^P < 0.001 (G4or G5 vs G3, G2 or G1) at respective times. All values are represented as mean ± SD, *n* = 10, animals in each group. Data were analyzed by one-way ANOVA, followed by Tukey-Kramer Multiple Comparisons Test. G1 (Control), G2((diabetic), G3((Standard control), G4 (GTE at 100 mg/ml), and G5 (GTE at 200 mg/ml).

### 3.4 Molecular changes in circulating miRNAs of diabetic skin wounds

**Fig. 3:**
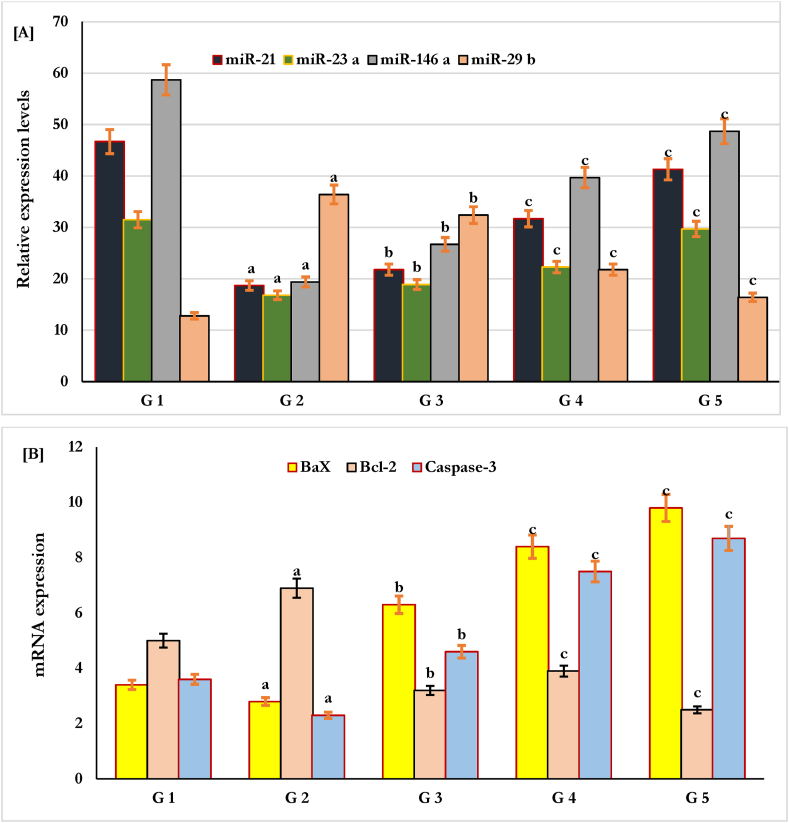
Effect of green tea hydroextract on differential Expression of MicroRNAs and apoptotic genes in treated and non-treated STZ-T1DM diabetic skin wounds.Differential Expression of MicroRNAs (miR-21, miR-23a, miR-146 a, and miR-29 b) [A], and apoptotic genes (Bax, Bcl-2, and Caspase-3) [B] in STZ-T1DM diabetic and non-diabetic skin wounds following treatment with green tea hydroextract at doses of 100 and 200 mg/ml for 21 days. G1 (Control), G2 ((diabetic), G3 ((Standard control), G4 (GTE at 100 mg/ml), and G5 (GTE at 200 mg/ml). ^a^P < 0.05 (G2 vs G1), ^b^P < 0.01 (G3 vs G1 or G2), and ^c^P < 0.001 (G4or G5 vs G3, G2 or G1).

The expression profiles of circulating miRNAs as molecular markers of wound healing were significantly estimated by PCR analysis in the tissues of T1DM diabetic and nondiabetic skin wounds treated with green tea extract, as shown in Figure (3). The results showed that the miRNAs miR-21, miR-23 a, miR-146 a, and miR-29b were differentially expressed in all skin tissues of T1DM diabetic and nondiabetic wounds (Fig. 3). The results in Figure 3A show that the expression of miR-21, miR-23a, and miR-146a was significantly (P < 0.05) downregulated (decreased), and miR-29b was significantly upregulated (increased; P < 0.05) in the skin wounds of T1DM diabetic rats (G2) compared to those in nondiabetic rats (G1) as a positive control.

When the skin wounds of T1DM diabetics were treated with GTE at doses of 100 mg/ml (G4) and 200 mg/ml (G5) for three weeks, as shown in Figure (3 A), the expression levels of targeted miRNAs were increased accordingly (Fig. 3A). The results showed that the expression levels of miR-21, miR-23a, and miR-146a significantly increased (upregulated, P < 0.001), and miR-29b expression levels significantly decreased (downregulated; P < 0.001) compared to those in diabetic non-treated-skin-wounds (G2) and intrasite gel as standard control drug (G3) (Fig. 3A). The results support that miRNAs are potentially considered molecular regulating cofactors in T1DM diabetic wounds.

### 3.5 Molecular changes in apoptotic genes of diabetic skin wounds

The expression of the apoptosis-related genes Bax, BCl-2, and caspase-3 in the skin wound tissues of diabetic and nondiabetic rats was identified by PCR analysis using specific primers (Fig. 3B). In skin wounds of T1DM diabetic rats (G2), the expression of the apoptosis-inducing genes Bax and caspase-3 was significantly reduced, and the expression levels of the anti-apoptotic Bcl-2 gene were significantly increased in diabetic tissues compared to those in control rats (G1) as a positive control (Fig. 3B). In addition, when diabetic rats were treated with intrasite gel as a standard control drug (G3), there was a significant increase in the expression levels of the apoptosis-inducing genes Bax and caspase-3 and a decrease in the expression levels of the Bcl-2 antiapoptotic gene compared to those in diabetic nontreated rats (G2), as shown in Figure 3A.

When green tea was applied as a healing support in diabetic wounds at doses of 100 mg/ml (G4) and 200 mg/ml (G5), as shown in Figure 3B, the results showed that the expression of the apoptosis-inducing genes Bax and caspase-3 significantly increased and the expression levels of the antiapoptotic Bcl-2 gene significantly decreased compared (p < 0.001) to those in untreated diabetic rats (G2) and control standard-treated rats (G3), as shown in Figure 3A.

The results suggested that green tea might accelerate the contraction and healing of wounds via the induction of cellular apoptosis. In T1DM diabetic wounds, the improvement in the parameters of wound healing, such as wound contraction, epithelialization, and scar formation, correlated positively with the expression of miRNAs, miR-21, miR-23a, miR-146a, and miR-29b, and apoptotic genes Bax and caspase-3 and negatively with the relative expression of the antiapoptotic Bcl-2 gene, diabetic controls, FBS, HbA1c, and C-peptide (Table 5).

**Table 5:** Correlation of wound healing paramters with diabetic variabels and expression of circulating miRNAs and apoptotic genes in diabetic skin wound tissues of rats treated with hydro extract of green tea for three weeks.

Variables	Wound closer (% contraction) (R)	**Scar formation (R)**	Epithelialization period (days) (R)
**Diabetes**			
**HbA1c**	−0.56^a^	−0.38^a^	−0.47^a^
**Insulin**	−0.65^a^	−0.74^a^	−0.25^a^
**C-peptide(ng/mL)**	−0.46^a^	−0.35^a^	−0.57^a^
**miRNAs concentrations**			
**miR-21**	0.54^b^	0.27^b^	0.38^b^
**miR-23a**	0.45^b^	0.34^b^	0.49^b^
**miR-146 a**	0.39^b^	0.58^b^	0.65^b^
**miR-29 b**	0.31^b^	0.47^b^	0.32^b^
**Apoptotic genes**			
**Bax**	0.72^c^	0.84^c^	0.71^c^
**Bcl-2**	−0.39^c^	−0.65^c^	−0.46^c^
**Caspase-3**	0.47^c^	0.54^c^	0.51^c^

Data are R (Spearman). HbA1C: glycated hemoglobin A1c, miR: microRNA, ^a^P < 0.05, ^b^P < 0.01, and ^c^P < 0.001.

In addition, a potential association between the expressed miRNAs and apoptosis was identified in diabetic wounds treated with green tea extract (Table 6). The data showed that the expression of the miRNAs miR-21, miR-23a, miR-146a, and miR-29b correlated positively with the relative expression of the apoptotic genes Bax and caspase-3 and negatively correlated with the antiapoptotic gene Bcl-2, supporting the molecular role of miRNAs in wound healing via apoptotic pathways (Table 6).

**Table 6:** Correlation between circulating miRNAs and apoptotic genes in diabetic skin wounds treated with hydro extract of green tea for three weeks.

Apoptotic genes	miRNAs concentrations			
	miR-21	miR-23 a	miR-146 a	miR-29b
Bax	0.49^b^	0.62^b^	0.27^c^	0.67^c^
Bcl-2	−0.28^b^	−0.37^b^	−0.36^c^	−0.58^c^
Caspase-3	0.21^b^	0.34^b^	0.19^c^	0.27^c^

Data are R (Spearman), miR: microRNA, ^a^P < 0.05, ^b^P < 0.01, and ^c^P < 0.001.

## 4 Discussion

In this study, the topical application of a hydro extract of green tea leaves at doses of 100 mg/ml and 200 mg/ml significantly accelerated diabetic wound healing in a shorter period (11–14 days).

The use of green tea extract (GTE) as an antidiabetic agent significantly improves diabetic profile parameters such as FBS, HbA1c, C-peptide, and insulin in all diabetic rats treated with green tea extract (GTE) at doses of 100 and 200 mg/ml. The antidiabetic activity of GTE might be related to the activity of polyphenols (GTPs) present in green tea. The data of this study showed that green tea has a considerable amount of EGCG (42.9 %), followed by EGC (12.0 %), EC (10.5 %), and ECG (7.1 %), with the lowest amount of caffeine (4.1 %).

Previous studies showed that the biological activities of green tea as an antidiabetic, antioxidant, and anti-inflammatory agent were related to the variations in phenolic and flavonoid contents, especially epigallocatechin content in GTE [36,49,[62], [63], [64]]. Supporting our results, in human patients with diabetes, the anti-diabetic control of green tea has been reported to proceed by preventing lipid accumulation, which consequently increases glycolipid metabolism and decreases the levels of blood sugar [65]. Additionally, a reduction in the levels of higher blood sugar was identified in animal models that consumed green tea or its active constituent, EGCG [66], and the improvement of blood glucose proceeded by stimulating glucose transporter 4 (GLUT4), which mediates glucose uptake in skeletal muscle [66]. In addition, administration of green or black tea for 14 weeks improves blood glucose without inhibiting α-glucosidase activity [[66], [67], [68]].

In different wounding models, herbal-based therapy using natural phenolic constituents of the plant was previously applied as an alternative potent remedy for the management of wound healing [[69], [70], [71], [72], [73]].

In the T1DM diabetic skin wound model, topical application of green tea extract (GTEs) at doses of 100 and 200 mg/ml was shown to improve wound healing by promoting and accelerating granulation, contraction, and epithelialization phases of wound healing within a short time (14 days) compared to that in the respective vehicle control standard. Consistent with our results, previous studies showed that when green tea or its active polyphenol (GTP) constituents were applied at higher concentrations, they accelerated the rate of wound healing by promoting angiogenesis by increasing fibroblast growth and collagen synthesis, reducing inflammation, and promoting the expression of vascular endothelial cell growth factor and angiopoietin-1 protein expression within the excision sites [53]. Then, wound closure and scar formation are accelerated, and complete epithelialization is performed in a short time [36,62,64,69,74].

The potential role of circulating microRNAs (miRNAs) as molecular regulators in diabetic wounds and the healing process was explored in this study. Our results showed that miRNAs were differentially expressed in the skin wound tissues of control, diabetic, and green tea-treated rats. miRNAs; miR-21, miR-23 a, miR-146 a significantly reduced and miR-29b significantly increased in the skin wounds of T1DM rats compared to those of normal nondiabetic rats. In contrast, in rats treated with green tea, the expression of miR-21, miR-23a, and miR-146 increased, and that of miR-29b was significantly reduced. The expression of miRNAs in the metabolism and in different phases of wound healing has been commonly studied, suggesting the potential role of miRNAs as regulators of growth, differentiation, and angiogenesis in the wound healing process in treated and nontreated wounds [[15], [16], [17], [18], [19], [20], [21],^75^,^76^]. Green tea leads to the upregulation of the expression of molecular miRNAs miR-21, miR-23 a, and miR-146a and the downregulation of miR-29b in diabetic wounds, which significantly promote keratinocyte migration, angiogenesis, the formation of new blood vessels, and a complete re-epithelialization process in a short time, as previously reported for each miRNA separately [[28], [29], [30],^77^,^78^]. Thus, in our study, treatment with green tea extract regulated the expression of miRNAs, especially miR-29b, to allow for better collagen synthesis and deposition as well as the formation of new blood vessels by angiogenesis [79,80]. These identified miRNAs controlled a set of genes, such as prolyl hydroxylase 1 and 2 (PHD1 and 2) genes, VEGF, and the expression of heat shock protein-47 (HSP47), which is needed for collagen synthesis and angiogenesis during skin wound healing [78,81]. In addition, molecular control of circulating microRNAs in promoting more collagen and fibronectin deposition as well as promoting the angiogenesis process significantly supports the activity of green tea as a regulator of diabetic wound healing [36]. It also signifies the functional role of circulating microRNAs as promoters of angiogenesis and vascular remodeling in diabetic wound healing [36].

Apoptosis is a natural process of programmed cell death that occurs in the body as a part of tissue turnover and regeneration. While excessive or insufficient apoptosis can both be detrimental to wound healing, the relationship between diabetes and apoptosis in this context is still an area of active research and debate. There is some evidence to suggest that increasing apoptosis may improve diabetic wound healing [[82], [83], [84]]. However, this is still a relatively new area of research, and more studies are needed to fully understand the relationship between apoptosis and diabetic wound healing. Thus, in this study, we tried to explore the role of apoptosis-related genes in the regulation of wound healing in diabetic rats. Our research results showed that the treatment of wounds of diabetic rats significantly increased the apoptosis process via a potential increase in the expression of apoptosis-inducing genes such as Bax and caspase-3 with a reduction or suppression of the antiapoptotic gene Bcl-2 in the skin wounds of diabetic rats treated with green tea extract.

Controlling apoptosis in the wound healing process is the most important mechanism needed in the healing process, especially in diabetes, whereas the increase in blood sugar significantly reduces or suppresses the apoptosis mechanism. In addition, the induction of cellular apoptosis in wounded tissues is needed for the elimination of unwanted cells and tissues during each phase of normal wound healing, particularly during the processes of inflammation, proliferation, and tissue remodeling [[85], [86], [87]]. Wound healing has also been shown to be associated with alterations in both apoptosis and cell proliferation [[79], [88], [89]]. Previously, supporting data also reported that treatment with green tea extract or its active polyphenolic compounds (GTPs) promotes the healing of skin by stimulating old keratinocytes and organizing cellular apoptotic genes [53,[80], [90], [91]].

Finally, the results also showed that the molecular miRNAs miR-21, miR-23a, miR-146a, and miR-29b were positively associated with the apoptotic-inducing genes Bax and caspase-3 and negatively associated with Bcl-2 antiapoptotic genes in the acceleration of wound contraction, epithelialization, and scar formation in diabetic wounds treated with green tea extract (GTE). This finding supports the molecular role of miRNAs in wound healing by regulating apoptotic pathways, as they are expressed as indicators of biological and physiological changes during healing mechanisms [[92], [93], [94]].

It was noted that the scientific evidence of using herbal medicine in treating the wounds of individuals with type 1 diabetes is limited, and further research is needed. In addition, some herbal remedies have been traditionally used for wound healing, but their effectiveness in the management of diabetic wounds requires more rigorous investigation. Therefore, it was recently revealed that miRNAs have emerged as critical players in impaired wound healing and could be targets for potential therapies for nonhealing wounds [95]. Moreover, in addition to their roles as regulators of gene expression, miRNAs have been demonstrated to mediate various skin pathologies, such as persistent inflammation or excessive scar formation [96,97].

The importance of miRNAs in the regulation of gene expression in most diseases such as cancer was extensively studied by gene regulatory networks (GRNs), whereas understanding the networks of any such gene by the use of molecular miRNAs will shed light on the mechanisms of many diseases such as cancer and of wounding healing mechanisms [98]. Recently, it was reported that gene regulatory networks (GRNs) affect numerous cellular processes and every process of life, and abnormalities in GRNs lead to serious diseases such as cancer. Thus, microRNAs (miRNAs) are one of the most studied gene regulatory mechanisms of GRNs within vital cells in most diseases, such as diabetes and cancer [[99], [100], [101]]. Thus, type 1 diabetes models for identifying the therapeutic role of green tea in the treatment of wounds based on the molecular basis, including cellular microRNAs and apoptosis, are the most important.

## 5 Strength of the study

The strengths of the study included the basic assessment of healing and control of molecular confounding variables such as miRNAs and apoptotic genes in T1DM diabetic wounds. In addition, no side effects or sensitivity to green tea ointment was observed in the green tea groups.

Moreover, this study focuses on catechins and other contained compounds in green tea extracts as improved standardization controls with higher reproducibility ability in healing effects that could be achieved in T1DM diabetic skin wounds. Thus, our results recommend that using a proper drug delivery system (DDS) that can deliver EGCG and other polyphenols present in green tea to the wound site could allow for drug delivery in a controlled manner, which is highly desirable for the treatment of diabetic wounds.

## 6 Limitations of the study

Although our study generally showed the importance of green tea in enhancing diabetic wound healing by controlling molecular changes in micro-RNAs and cellular apoptosis genes, the lack of application of green tea on nondiabetic wounds as well as long-term follow-up leads to difficulty in predicting the exact mechanism underlying the role miRNAs in association with apoptotic genes on diabetic wound healing. Therefore, our results could be interpreted as preliminary findings and further studies are needed to understand the potential association of these biological parameters with the wound healing process in diabetic wounds.

## 7 Conclusion

Green tea ointment appears to be effective in improving T1DM diabetic wound healing in this study. The results showed that tissue remodeling and scar formation with less scar width at wound closure were identified in green tea-treated diabetic wounds. Green tea shows high potential in wound healing activity by controlling cellular apoptosis and the molecular expression of circulating microRNAs: miR-21, miR-23a, miR-146a, and miR-29b. Thus, to establish a future model for the treatment of diabetic wounds, further studies are needed to understand the potential association of these biological parameters with the wound-healing process in diabetic wounds. In addition, investigating the pattern of molecular miRNAs and their correlation with the progression of diabetic wound healing might provide valuable information, that could be fed into gene regulatory networks (GRNs), for understanding the molecular mechanism of gene regulation in wound healing as a whole and the molecular basis of healing in individuals with type 1 diabetes.

## Funding statement

This project was funded by the **Deputyship for Research & Innovation,** **Ministry of Education** **in Saudi Arabia. (IFKSURC-1-0601)**.

## Data availability statement

All data generated or analyzed during this study are presented in the manuscript. Please contact the corresponding author for access to the data presented in this study.

## CRediT authorship contribution statement

**Hadeel A. Al-Rawaf:** Conceptualization, Writing – original draft. **Sami A. Gabr:** Conceptualization, Data curation, Formal analysis, Investigation, Methodology, Project administration, Supervision, Validation, Writing – original draft, Writing – review & editing. **Ahmad H. Alghadir:** Writing – original draft, Writing – review & editing.

## Declaration of competing interest

The authors declare that they have no known competing financial interests or personal relationships that could have appeared to influence the work reported in this paper.
